# Ultrasonic Sensitivity of Strain-Insensitive Fiber Bragg Grating Sensors and Evaluation of Ultrasound-Induced Strain

**DOI:** 10.3390/s101211248

**Published:** 2010-12-08

**Authors:** Hiroshi Tsuda, Kenji Kumakura, Shinji Ogihara

**Affiliations:** 1 National Institute of Advanced Industrial Science & Technology/AIST Tsukuba Central 2, Tsukuba, 305-8568, Japan; 2 Department of Mechanical Engineering, Faculty of Science and Technology, Tokyo University of Science/2641 Yamazaki, Noda, 278-8510, Japan; E-Mails: kumakura-kenji@aist.go.jp (K.K.); ogihara@rs.noda.tus.ac.jp (S.O.)

**Keywords:** fiber Bragg grating, ultrasonic sensitivity, strain, ultrasound detection

## Abstract

In conventional ultrasound detection in structures, a fiber Bragg grating (FBG) is glued on or embedded in the structure. However, application of strain to the structure can influence the sensitivity of the FBG toward ultrasound and can prevent its effective detection. An FBG can work as a strain-insensitive ultrasound sensor when it is not directly glued to the monitored structure, but is instead applied to a small thin plate to form a mobile sensor. Another possible configuration is to affix an FBG-inscribed optical fiber without the grating section attached to the monitored structure. In the present study, sensitivity to ultrasound propagated through an aluminum plate was compared for a strain-insensitive FBG sensor and an FBG sensor installed in a conventional manner. Strains induced by ultrasound from a piezoelectric transducer and by quasi-acoustic emission of a pencil lead break were also quantitatively evaluated from the response amplitude of the FBG sensor. Experimental results showed that the reduction in the signal-to-noise ratio for ultrasound detection with strain-insensitive FBG sensors, relative to traditionally-installed FBG sensors, was only 6 dB, and the ultrasound-induced strain varied within a range of sub-micron strains.

## 1. Introduction

In a fiber Bragg grating (FBG), the core of a single-mode optical fiber is subjected to periodic modulation of its refractive index. This creates a narrowband reflective filter. The reflected wavelength is called the Bragg wavelength and is influenced by the strain and temperature applied to the FBG. An FBG with a Bragg wavelength of 1.55 μm has sensitivities to strain and temperatures of 14 pm/K and 1.2 pm/με, respectively [1]. Ultrasound impinging on an FBG induces a subtle Bragg wavelength shift because ultrasound induces a small strain change in the FBG. This shift in the Bragg wavelength can be detected by a demodulation technique employing a tunable laser. In an ultrasonic sensing system with a tunable laser, the laser is tuned to a wavelength where the gradient of the FBG reflective spectrum is steep, such as the wavelength at which the FBG reflectivity is reduced by half [2]. The change in the intensity of light reflected from the FBG corresponds to the amplitude of ultrasound exerted on the FBG. A photodetector is used to measure the intensity of light reflected from the FBG.

**Figure 1.**
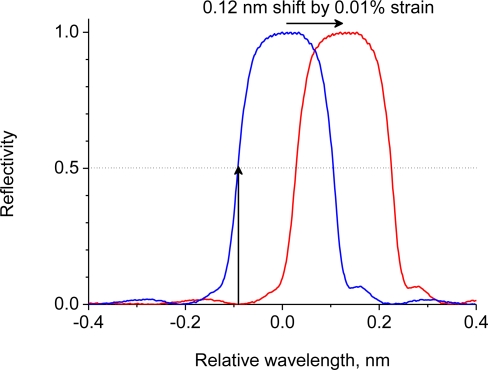
A typical reflection spectrum of a 10-mm-long fiber Bragg grating.

In previous studies on ultrasound detection, FBGs were typically glued to or embedded in the structures to be monitored. Direct attachment of the FBG enables sensitive ultrasound detection. However, the reflective spectrum shifts with the strain applied to the structure [3–7]. Figure 1 shows the reflective spectrum of a 10-mm-long FBG that is commonly used for detecting ultrasound. In this setup, the spectrum shifts by 0.12 nm when the FBG is subjected to 0.01% strain. If the lasing wavelength is originally set to 50% of the reflective spectrum, a strain of just 0.01% can reduce the reflectivity at the lasing wavelength to zero. At this point, the FBG sensor would not be able to perform ultrasound detection. Furthermore, ultrasound detection by an affixed FBG suffers even more under a non-uniform strain distribution. For example, matrix cracking in composite materials causes a non-uniform strain distribution. Non-uniform strain distribution along the FBG deforms the reflection spectrum and may even split the spectrum into multiple peaks [8,9]. This distorted reflection spectrum results in a sensor output signal response that is inconsistent with the ultrasonic vibration impinging on the FBG [3].

Strain-insensitive FBG ultrasound sensing systems that incorporate broadband light sources and optical filters used as demodulators have been proposed [10,11]. These systems employ optical filters that feature periodical optical characteristics such as arrayed waveguide gratings and Fabry-Perot filters. Using these systems, the Bragg wavelength shift induced by ultrasound can be detected by monitoring the change in intensity of light transmitted through the filters, irrespective of the Bragg wavelength. However, these systems still have a critical drawback; the sensitivity toward ultrasonic vibration is too low to acquire a sufficient amplitude response without averaging the response signal. Thus, systems with broadband light sources and optical filters are unlikely to detect low amplitude acoustic emissions (AEs).

Two types of strain-insensitive FBG ultrasound sensors with laser-based light sources have been proposed. In the first, an FBG-inscribed optical fiber without the grating section is attached to the monitored material. Ultrasound propagating in the material travels along the optical fiber via the point of contact; this subsequently reaches and impinges on the FBG [12,13]. One author has reported the continuous measurement of AE using a strain-insensitive FBG sensor under varying strain conditions. Acoustic emissions have been detected continuously during a pressure test in which a carbon-fiber filament-wound vessel was pressurized to 1% strain [14].

In the second configuration for a strain-insensitive sensor, an FBG is attached on a small thin plate, and the plate is placed on the monitored material. This configuration permits mobility of the FBG sensor. Ultrasound propagating in the monitored material penetrates the thin plate and then impinges on the connected FBG [15,16]. One author has reported that the location of a fatigue crack tip could be positioned precisely using a mobile FBG sensor, in which an FBG was glued on an acrylic plate [17].

A strain-insensitive FBG sensor can detect ultrasound irrespective of the strain applied to a monitored structure because the grating section is separate from the structure. Furthermore, ultrasound can be easily detected at any place using a mobile strain-insensitive FBG sensor. Despite the advantages of strain-insensitive FBG sensors over conventionally-glued FBG sensors, their ultrasonic sensitivity has not yet been adequately investigated. In this study, the sensitivity of a strain-insensitive sensor was evaluated and compared with that of an FBG installed in a conventional manner. Furthermore, strains induced by ultrasound vibration and quasi-AE were quantitatively evaluated from the FBG sensor responses.

## 2. Influence of Wave Mode on Ultrasonic Sensitivity of the FBG Sensors

A 400 × 200 × 1-mm aluminum plate was used as a specimen for ultrasound propagation. Ultrasound vibrations propagating in a thin plate are known as Lamb waves. There are two modes of propagation: the symmetrical mode and the asymmetrical mode. The influence of wave mode on ultrasonic sensitivity of an FBG sensor was the first parameter evaluated in this study. A shear wave transducer with a central frequency of 250 kHz (Panametrics, V150) and a longitudinal wave transducer with a central frequency of 180 kHz (Panametrics, X1019) were used to generate symmetrical mode and asymmetrical-mode waves, respectively. A pulse of 375 V (peak voltage) was used as the incident signal to the transducer. The resulting ultrasound was in the form of the fundamental symmetrical-mode wave (S_0_) or the fundamental asymmetrical-mode wave (A_0_) because the product of the plate thickness and the ultrasound frequency was lower than 1 MHz·mm [18]. A 10-mm-long FBG with a Bragg wavelength of 1,550 nm was glued on the aluminum plate 100 mm away from the piezoelectric transducer.

**Figure 2.**
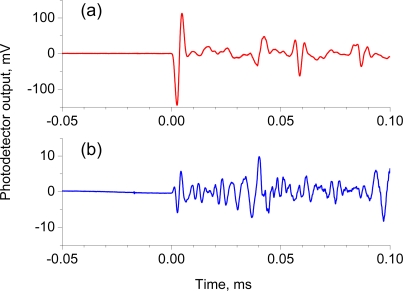
512-time-averaged FBG sensor response to Lamb waves: **(a)** Response to S_0_ waves, **(b)** Response to A_0_ waves.

Figure 2(a,b) shows the 512-time-averaged response signal to the S_0_ and A_0_ Lamb waves, respectively. A well-defined one-cycle sinusoidal response with amplitude ranging from −145 to 113 mV was found in the response to the S_0_ wave. The A_0_ wave, on the other hand, created a weak response around 1 mV followed by a one-cycle sinusoidal response at ±6 mV and then a continued weak response at a few mV signal level. The continued small response resulted from the dispersive characteristics of A_0_ waves [18]. The response to S_0_ waves was more than twenty times higher in amplitude compared to the response to A_0_ waves. The FBG sensor attached on a thin plate proved to be more sensitive to symmetrical-mode waves. Thus, the following experiments were performed using symmetrical-mode waves exclusively.

## 3. Ultrasound Sensitivities of a Strain-insensitive FBG Sensor and Conventionally-glued FBG Sensor

**Figure 3.**
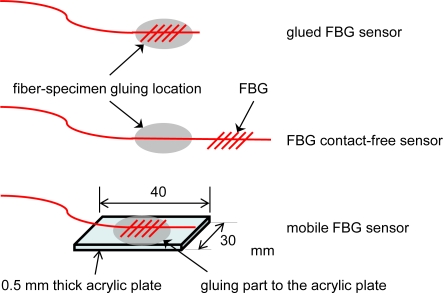
Schematics illustrating the configurations of FBG sensors employed in the present study.

The influence of FBG sensor configuration on ultrasound sensitivity was investigated using three different FBG sensors, as shown in Figure 3. The first sensor, in which an FBG is glued on the specimen, is called a glued FBG sensor. This installation has been commonly employed in ultrasound detection with FBGs. The second sensor, in which an FBG-inscribed optical fiber minus the grating section is glued to the specimen, is called an FBG contact-free sensor. The third sensor, in which an FBG is glued on a small thin plate, is called a mobile FBG sensor. The last two sensors are strain-insensitive FBG sensors. A 10-mm-long FBG with a Bragg wavelength of 1,550 nm was re-glued in the different configurations for a series of experiments.

**Figure 4.**
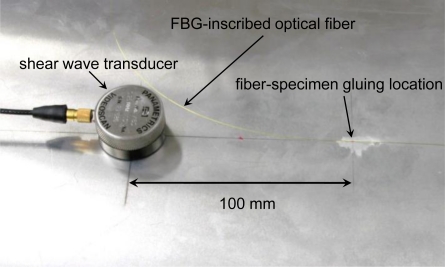
Photograph showing the installation of a glued FBG sensor and an FBG contact-free sensor.

**Figure 5.**
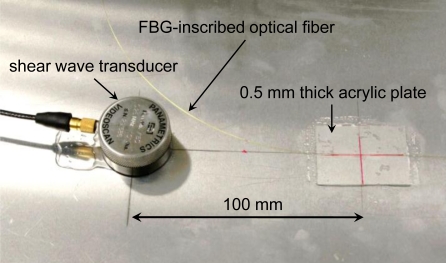
Photograph showing the installation of a mobile FBG sensor.

Figure 4 shows the experimental setup for the glued FBG sensor and the FBG contact-free sensor. Figure 5 shows the experimental setup for the mobile FBG sensor. The distance between the FBG and the ultrasound transducer was 100 mm for the glued and mobile FBG sensors. The span between the FBG and the ultrasound transducer was 150 mm for the FBG contact-free sensor because the FBG was 50 mm away from the glued part of the optical fiber. The S_0_ wave was generated by a shear wave transducer (Panametrics, V150) using a 375-V pulse (peak voltage).

**Figure 6.**
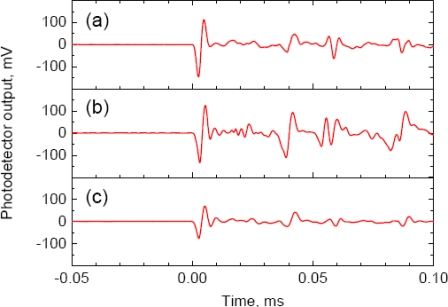
Responses to ultrasound detected by **(a)** a glued FBG sensor, **(b)** an FBG contact-free sensor, and **(c)** a mobile FBG sensor.

The 512-time-averaged ultrasonic responses of the three different sensors are shown in Figure 6. Table 1 lists the root-mean-square (RMS) value of noise appearing before the ultrasonic response (N_rms_), the amplitude of the initial response (V_pp_), and the signal-to-noise ratio (SNR) calculated from Equation (1):

(1) $$\text{SNR}=20\text{log}\frac{V_{\text{pp}}}{N_{\text{rms}}}$$

The noise level of the FBG contact-free sensor approximately doubled compared with the other FBG sensors with fixed grating sections. The higher noise level in the FBG contact-free sensor could be attributed to environmental perturbation. This is because the grating was not structurally fixed and was therefore prone to external disturbance. There was, however, little difference in the response amplitudes between the FBG contact-free sensor and the glued FBG sensor. This shows that the ultrasonic vibration experienced little attenuation along the 50-mm optical fiber before reaching the grating section in the FBG contact-free sensor.

The response amplitude of the mobile FBG sensor was reduced by around half compared with the glued FBG sensor, though the two sensors had almost the same noise level. A viscous gel for shear wave transducers was used as a coupler between the acrylic plate of the mobile FBG sensor and the specimen. It can be inferred that the ultrasonic vibration was attenuated in the viscous couplant before penetrating the acrylic plate and impinging on the FBG.

The ultrasound response of the glued FBG sensor had the highest SNR of 69 dB. The two strain-insensitive FBG sensors had almost the same SNR and the SNRs were about 6 dB lower than the glued FBG sensor. The SNR of the ultrasonic response detected by a piezoelectric transducer, which was identical to the transducer used for emitting ultrasound, was evaluated for reference. Its SNR was 75 dB, which was 6 dB higher than that of the glued FBG sensor.

## 4. Quantitative Evaluation of Ultrasound-Induced Strain

**Figure 7.**
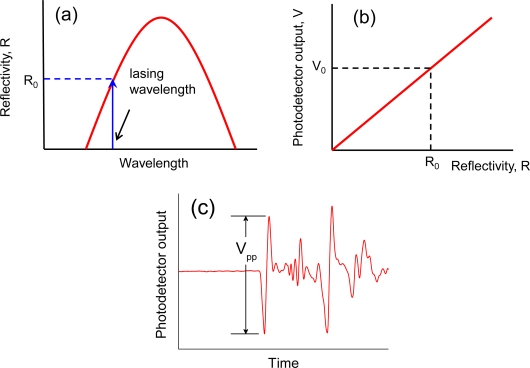
Schematics illustrating the evaluation of strain induced by an ultrasonic vibration. **(a)** The reflection spectrum of an FBG sensor. **(b)** The relation between the photodetector output and the reflectivity of the FBG sensor at the lasing wavelength. **(c)** An example of an obtained photodetector output signal.

Strain induced by an ultrasonic vibration was evaluated using a calculation method proposed by Betz *et al.* [3]. For a laser launched into an FBG, R_0_ and V_0_ are the reflectivity of the FBG at the lasing wavelength and the photodetector output, respectively, as shown in Figure 7(a,b). The reflection spectrum oscillates synchronously with the ultrasonic vibration impinging on the FBG. When the FBG is subjected to an ultrasound-induced strain fluctuation, ε(t), the reflectivity at the lasing wavelength as a function of time R(t), can be written as follows:

(2) $$R(t)=R_{0}+\frac{dR}{dɛ}\cdot ɛ(t)$$

As the photodetector output varies in proportion to the reflectivity at the lasing wavelength, as shown in Figure 7(b), the photodetector output as a function of time, V(t), is given by Equation (3):

(3) $$V(t)=\frac{V_{0}}{R_{0}}\cdot R(t)$$

Substituting Equation (3) into Equation (2) yields Equation (4):

(4) $$ɛ(t)=\frac{R_{0}}{V_{0}}[V(t)-V_{0}]\cdot {\left(\frac{dR}{dɛ}\right)}^{-1}$$

When the photodetector output signal responding to an ultrasonic vibration is given as in Figure 7(c), the change in strain applied to the FBG by the ultrasonic vibration, Δε, is given by Equation (5):

(5) $$\Delta ɛ=\frac{R_{0}}{V_{0}}\cdot V_{pp}\cdot {\left(\frac{dR}{dɛ}\right)}^{-1}$$

where V_pp_ is the amplitude of the initial response and the following relation is applied:

(6) $$\frac{dR}{dɛ}=\frac{dR}{d\lambda }\cdot \frac{d\lambda }{dɛ}$$

The first term, dR/dλ, is the slope of the FBG reflection spectrum at the lasing wavelength. This value can be assessed from the reflection spectrum, as measured with an optical spectrum analyzer. The second term, dλ/dε, is the strain sensitivity of the Bragg wavelength shift and is given as 1.2 pm/με for a 1.55 μm Bragg grating.

The values of dR/dλ and the evaluated ultrasound-induced strain are listed in Table 2. Both the glued FBG sensor and the FBG contact-free sensor were subjected to the same strain change of ±0.7 με. The resulting Bragg wavelength shift was calculated to be ±0.84 pm because the strain sensitivity of the Bragg wavelength shift was 1.2 pm/με. On the other hand, the ultrasound-induced strain and the corresponding Bragg wavelength shift for the mobile FBG sensor were evaluated to be ±0.5 με and ±0.6 pm, respectively. The couplant between the mobile sensor and the specimen acted as a strain buffer, reducing the strain applied to the FBG affixed to the mobile sensor.

The ultrasound generated by a piezoelectric transducer with a pulse of 375 V corresponds to an AE with high amplitude. Most AEs that accompany microscopic failures of materials have weaker amplitudes than the ultrasonic vibrations produced in this study. Thus, the change in strain induced by most AEs would be in the sub-micron strain range, and the corresponding Bragg wavelength shift would be in the sub-picometer range.

The strain induced by A_0_ waves was estimated from the response shown in Figure 2 to be ±0.02 με. This strain is quite small compared with that induced by S_0_ waves, ±0.7 με. The strain evaluated herein corresponds to the axial strain of the FBG, which is identical to the in-plain strain of the specimen. This result agrees with the fact that in-plain displacement induced by asymmetrical-mode waves is quite small compared with symmetrical-mode waves [19].

## 5. FBG Sensor Response to Pencil Lead Break

A pencil lead break has been employed as a quasi-AE signal for the sensitivity measurement of AE sensors, as well as for calibration of the AE source location [19]. The intensity of the quasi-AE generated by breaking a pencil lead was compared with that of the ultrasound generated from a piezoelectric sensor. The aluminum plate employed in the aforementioned experiments was used as a test specimen. The pencil-lead break test was performed in accordance with the Japanese Society for Non-Destructive Inspection Standards (NDIS) 2110 [20]. A 3-mm-long pencil lead was broken on the edge of the aluminum plate to generate S_0_-dominated Lamb waves, and the response was acquired by the glued FBG sensor used in the previous experiments. The distance between the lead breaking point and the glued FBG sensor was 100 mm.

**Figure 8.**
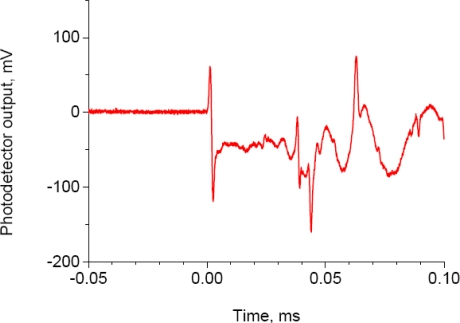
A typical glued FBG sensor response to a pencil lead break.

An example of the glued FBG sensor response to quasi-AE by a lead break is shown in Figure 8. The FBG sensor responded as an asymmetrical one-cycle signal ranging from 60 to −120 mV. The feature of the response was different from the response generated by a piezoelectric transducer, as shown in Figures 2 and 6, which exhibited a nearly symmetrical sinusoidal response. The average strain evaluated from ten pencil lead break tests ranged from 0.44 to −0.91 με, and the standard deviations of both the tensile and compressive strains were 0.1 με. The change in strain induced by lead breaks was estimated to be 1.35 με. This is very close to 1.4 με, which was the strain change induced from the ultrasonic vibration that was excited by a piezoelectric transducer under the present experimental conditions.

## 6. Conclusions

The sensitivity of strain-insensitive FBG sensors toward ultrasonic vibrations and the strain induced by ultrasonic vibrations were quantitatively evaluated in this study. The following conclusions may be drawn from these results:

1. An FBG sensor affixed to the surface of a thin plate had higher sensitivity to symmetrical-mode waves than asymmetrical-mode waves. This is because symmetrical-mode waves result in greater in-plane displacement, to which the Bragg wavelength of the FBG shifts in a sensitive manner.
2. The FBG contact-free sensor had a higher noise level because it was prone to environmental perturbations. The mobile FBG sensor had a smaller response signal because the couplant between the specimen and the movable plate to which the FBG was attached worked as an ultrasound attenuator. The SNR of the response signal detected by strain-insensitive FBG ultrasonic sensors was reduced by around 6 dB compared with the FBG affixed to the specimen.
3. The strain induced by ultrasound was quantitatively evaluated from the FBG sensor responses. The change in strain induced by pencil-lead breaks was very close to the ultrasound-induced strain generated by a piezoelectric transducer, to which a pulse of 375 V was applied. The strain change resulting from AEs accompanying a microscopic failure of materials would be in the sub-micron strain range and the resulting Bragg wavelength shift would be in the sub-picometer range.

## Figures and Tables

**Table 1.** Characteristics of FBG sensor responses to ultrasound.

Type of sensor	RMS value of noise N_rms_ (mV)	Response amplitude V_pp_ (mV)	SNR (dB)
Glued FBG sensor	0.10	282	69
FBG contact free sensor	0.19	256	63
Mobile FBG sensor	0.12	153	62
Piezoelectric sensor	-	-	75

**Table 2.** The gradient of the FBG reflection spectrum and evaluated strain change induced by ultrasound.

Type of sensor	dR/dλ (pm^−1^)	Δε (με)
Glued FBG sensor	30.0E-3	± 0.7
FBG contact free sensor	27.4E-3	± 0.7
Mobile FBG sensor	20.8E-3	± 0.5
